# Supplementary material for: Gender Differences in Obstructive Sleep Apnea: A Preliminary Clinical and Polysomnographic Investigation
Source: Neurol Int. 2025 May 29;17(6):85. doi: 10.3390/neurolint17060085 (PMC12195948; doi:10.3390/neurolint17060085)
Supplement: Supplementary file 1 [file neurolint-17-00085-s001.zip › neurolint-3622734-supplementary.pdf]

S1: Detailed Description of the Neuropsychological Test Battery Administered

Mini Mental State Examination (MMSE)

The Mini-Mental State Examination (MMSE) includes 11 questions or tasks divided across various cognitive areas: time orientation, place orientation, repeating three words, attention and arithmetic, recalling the three words, praxis, language skills, and visual-spatial construction. The maximum score is 30, which serves as an indicator of a person's cognitive functioning, assessed through direct observation while completing the tasks. A score below 24 is commonly recognized as the threshold suggesting cognitive impairment.

Measso, G., Cavarzeran, F., Zappalà, G., Lebowitz, B.D., Crooke, T.H., Pirozzolo, F.J., et al., 1991. The Mini-Mental state examination. Normative study of an Italian random sample. Dev. Neuropsychol. 9, 77-85

Digit Span forward and backward

The digit-span task assesses the capacity of working memory for storing numbers. In this task, participants hear a sequence of digits (such as "8, 3, 4") and must immediately repeat them. If successful, they are given a longer sequence (e.g., "9, 2, 4, 0"). The longest sequence a person can accurately recall determines their digit span. In the forward version, participants repeat the digits in the same order, while in the backward version, they must recall the digits in reverse order.

Orsini, A., Grossi, D., Capitani, E., Laiacona, M., Papagno, C., Vallar, G., 1987. Verbal and spatial immediate memory span: normative data from 1355 adults and 1112 children. Ital. J. Neurol. Sci. 8, 539-548

Spinnler, H., Tognoni, G., 1987. Standardizzazione e taratura italiana di test neuropsicologici. Ital. J. Neurol. Sci. 8.

Mondini, S., Mapelli, D., Vestri, A., Bisiacchi, P.S., 2003. L'esame neuropsicologico breve. Raffaello Cortina, Milano

Corsi block-tapping Test

The Corsi Block-Tapping Test is a psychological assessment used to measure visuospatial short-term and working memory. The task involves a board with nine blocks arranged in fixed, pseudorandom positions. Each block is numbered, but only the experimenter can see the numbers. During the test, the experimenter taps a sequence of blocks, and the participant must reproduce the sequence in the same order. As the test progresses, the sequences become longer. The final score corresponds to the longest sequence the participant can accurately recall.

Kessels, R.P., van den Berg, E., Ruis, C., Brands, A.M., 2008. The backward span of the Corsi Block-Tapping Task and its association with the WAIS-III Digit Span. Assessment 15, 426-434.

Orsini, A., Grossi, D., Capitani, E., Laiacona, M., Papagno, C., Vallar, G., 1987. Verbal and spatial immediate memory span: normative data from 1355 adults and 1112 children. Ital. J. Neurol. Sci. 8, 539-548

Spinnler, H., Tognoni, G., 1987. Standardizzazione e Taratura Italiana di test neuropsicologici. Ital. J. Neurol. Sci. 8.

Rey's List: learning, recall and recognition

The examiner reads a list of 15 words, one word per second, and repeats this process five times. After each reading, the participant is asked to recall as many words as possible, in any order. The total number of words recalled across the five trials represents the Immediate Recall score (ranging from 0 to 75). After a 15-minute break during which the participant

completes nonverbal tasks, they are asked to recall the words again, which constitutes the Delayed Recall score (ranging from 0 to 15). In the Recognition phase, the examiner reads a new list that includes the original 15 words from Rey's list, and the participant must identify the words they remember.

Carlesimo, G.A., Caltagirone, C., Gainotti, G., 1996. The Mental Deterioration Battery: normative data, diagnostic reliability and qualitative analyses of cognitive impairment. The Group for the Standardization of the Mental Deterioration Battery. Eur. Neurol. 36, 378-384

Raven's Progressive Matrices

Raven's Progressive Matrices is a visual test designed to assess abstract reasoning abilities. It consists of 36 multiple-choice items, organized into three sets of 12. Each item features a pattern or image with one missing piece in the upper section. Below the image, there are six possible options, and only one correctly completes the pattern. Participants must select the fragment that best fits the missing part. The total score is based on the number of correct answers out of 36.

Spinnler, H., Tognoni, G., 1987. Standardizzazione e taratura italiana di test neuropsicologici. Ital. J. Neurol. Sci. 8.

Attentive Matrices

The Attentive Matrices Test is a reliable tool for evaluating both selective and sustained attention. It involves a series of number grids, where the participant is asked to scan through the numbers and identify a specific target number, which may range from one to three digits.

Spinnler, H., Tognoni, G., 1987. Standardizzazione e Taratura Italiana di test neuropsicologici. Ital. J. Neurol. Sci. 8.

Verbal Fluency with Phonemic cues

The participant is asked to generate as many words as possible that begin with a specific letter of the alphabet. This task is repeated for three different letters, with each round lasting 60 seconds. The total score is the sum of all correctly produced words across the three trials.

Verbal Fluency with Semantic cues

The participant is asked to name as many words as possible that belong to a specific semantic category, such as animals or fruits. This task is performed for three different categories, each within a 60-second time limit. The total score is the sum of all correctly produced words across the three categories.

Novelli, G., Papagno, C., Capitani, E., Laiacona, M., Vallar, G., Cappa, S.F., 1986. Tre test clinici di ricerca e produzione lessicale. Taratura su soggetti normali. Archivio di Psicologia Neurologia e Psichiatria 47, 477-506

Token Test

The Token Test uses tokens that differ in shape (circles and rectangles), size (large and small), and color (white, red, yellow, green, and black). The test is divided into six parts. In the first five, instructions are given using simple sentence structures and gradually increase in verbal memory demand. In the sixth part, the instructions become syntactically more complex, relying heavily on grammatical elements. The total score, ranging from 0 to 36, is based on the number of commands the participant correctly follows.

De Renzi, E., Faglioni, P., 1978. Normative data and screening power of a shortened version of the Token Test. Cortex 14, 364-381

Copy of Rey-Osterrieth complex figure

The Rey-Osterrieth Complex Figure Test (ROCF) is a widely used neuropsychological assessment for evaluating visuospatial constructional abilities. The test consists of 18 components, with a maximum score of 36 for each of the two tasks (direct and delayed copying). Points are awarded based on the accuracy of reproduction: 2 points for correct reproduction, 1 point for a distorted or incomplete element that is placed correctly, or a complete element placed incorrectly; 0.5 points are given for a distorted or incomplete element placed poorly. A score of 0 is assigned if the element is missing or unrecognizable. To administer the ROCF, a figure is shown to the participant, who is then asked to copy it as accurately as possible.

Caffarra, P., Vezzadini, G., Dieci, F., Zonato, F., Venneri, A., 2002. Rey-Osterrieth complex figure: normative values in an Italian population sample. Neurol. Sci. 22, 443-447
